# Evolutionary conservation of a regulative pathway of erythropoiesis in Poikilothermic vertebrates

**DOI:** 10.1038/s41598-022-06617-6

**Published:** 2022-02-28

**Authors:** Rosa Manca, Monia Delia, Marianna Abate, Silvia Zappavigna, Sergio Papa, Chester Glomski, Alessandra Pica

**Affiliations:** 1 Department of Biology, University of Naples “Federico II”, Naples, Italy; 2 Department of Basic Medical Sciences, Neuroscience and Sense Organs, University of Bari “Aldo Moro”, Bari, Italy; 3 Department of Precision Medicine, University of Campania “L. Vanvitelli”, Naples, Italy; 4 Department of Basic Medical Sciences, Neurosciences and Sense Organs, University of Bari “Aldo Moro”, Bari, Italy; 5 Department of Biology and Evolution of Marine Organisms, Zoological Station of Naples “A. Dohrn”, Naples, Italy; 6 Department of Pathology and Anatomical Sciences, Jacobs School of Medicine, State University of New York, Buffalo, NY USA

**Keywords:** Biochemistry, Histocytochemistry, Immunochemistry, Proteins, Haematopoietic stem cells, Evolutionary developmental biology

## Abstract

Apoptosis, programmed cell death, plays a central role in haematopoiesis. Mature erythrocytes of non-mammalian vertebrates maintain a permanent nucleus; these cells can undergo apoptosis (eryptosis), as do other somatic cells of a given non-mammalian vertebrate. In this study, we have investigated the expression and subcellular distribution of Bcl-2, Bcl-X_L_ and Bax proteins in the maturation phases and after X-ray irradiation of nucleated erythrocytes of *Torpedo marmorata* and *Caretta caretta* and the effect of X-ray irradiation on nucleated circulating erythrocytes of *Torpedo marmorata.* The cellular distribution of proteins was detected in erythrocytes by using immunocytochemistry at light microscopy and immunoelectron microscopy. The electrophoretic separation and immunoblotting of pro- and anti-apoptotic proteins of immature and mature erythroid cells was performed too, after X-ray irradiation of torpedoes. The results of the immunocytochemical analyses show an increase, in the expression level of Bax in mature as compared to young erythrocytes and a corresponding decrease of Bcl-2 and Bcl-X_L_. This maturation pattern of Bax, Bcl-2 and Bcl-X_L_ was abrogated in X-ray irradiated torpedo erythrocytes. On the basis of these observations, Bax, Bcl-2 and Bcl-X_L_ seems to play a role in the erythropoiesis of *Torpedo marmorata* Risso and in *Caretta caretta*. In conclusion, the same apoptotic proteins of somatic cells appear to be conserved in circulating nucleated erythrocytes thus suggesting to play a role in the maturation of these cells.

## Introduction

Mature circulating erythrocytes of non-mammalian vertebrates maintain the nucleus and organelles including mitochondria. These cells can undergo apoptosis (eryptosis) as do other somatic cells^1^.

Eryptosis of circulating nucleated erythrocytes^2^ of poikilothermic vertebrates, such as the elasmobranch *Torpedo marmorata* Risso and the sea turtle *Caretta caretta,* constitutes a useful model to study whether the maturation process of nucleated erythrocytes in circulation and the regulation of their apoptotic mechanism is similar to that one of non-erythroid somatic cell in mammalian vertebrates^3^.

Three maturation stages of red blood cells are identifiable in circulating blood of torpedoes^4^. The first is represented by basophilic erythroblasts, in which 50–60 small round mitochondria encircle the nucleus. These cells are spherical and present a large nucleus with small amounts of condensed chromatin and a nucleolus. The cytoplasm is filled with ribonucleoproteins, mitochondria and vacuoles. In a further maturation progression, the cells lose their round configuration becoming elliptical in shape and condensation of chromatin increases. In electron micrography the cytoplasm shows mitochondria, abundant ribosomes, profiles of rough endoplasmic reticulum, a small Golgi complex, vesicles and vacuoles. The second maturation stage, the acidophilic erythroblasts, representing around 17% of circulating erythrocytes, has larger, slightly elongated mitochondria, distributed near the nucleus, in number ranging from 40 to 50 per cell. The nuclear electron density is increased while the quantity of ribosomes is diminished. The mature erythrocytes, the third stage, have much larger but fewer mitochondria, than the two previous stages, mainly adjacent to the two poles of the ellipsoidal nucleus^5,6^.

Circulating erythropoiesis is identifiable in the blood of the loggerhead turtles. The youngest recognizable immature precursor of the erythrocyte is the basophilic erythroblast, a spherical cell with centrally located round nucleus surrounded by a relatively narrow band of basophilic cytoplasm. With continued maturation the cell evolves into an intermediate form, the acidophilic erythroblast, which presents a flattened ellipsoidal configuration: the cytoplasm is completely acidophilic like that of mature erythrocyte, due to the accumulation of hemoglobin, while the nucleus is oval and the chromatin is less compact than in mature red cell. The loggerhead reticulocyte is a further developed cell and it is the immediate antecedent of the mature erythrocyte. It is identified by the detection of a small amount of residual cytoplasmic RNA^7^. The B cell lymphoma-2 protein family plays a key role in human apoptosis^8^. Anti- apoptotic members, such as Bcl-2 and Bcl-X_L_, inhibit apoptosis of haematopoietic progenitor cell counteracting apoptosis activators, such as Bax. Intrinsic apoptosis of mature erythrocyte can be affected by diverse stimuli including genotoxic damage by X-ray exposure^9,10^. Pica et al*.*^11^*,* have shown that in elasmobranchs the higher doses of X-rays than in mammals have to be used to cause haemopoietic depletion. In the present study, we have detected the expression and subcellular distribution of Bcl-2, Bcl- X_L_ and Bax proteins in the maturation phases of *Torpedo marmorata* and *Caretta caretta* and the effect of X-ray irradiation on nucleated circulating erythrocytes of *Torpedo marmorata*.

## Results

### Bcl-2, Bcl-X_L_ and Bax expression regulation during erythropoiesis

**Figure 1:**
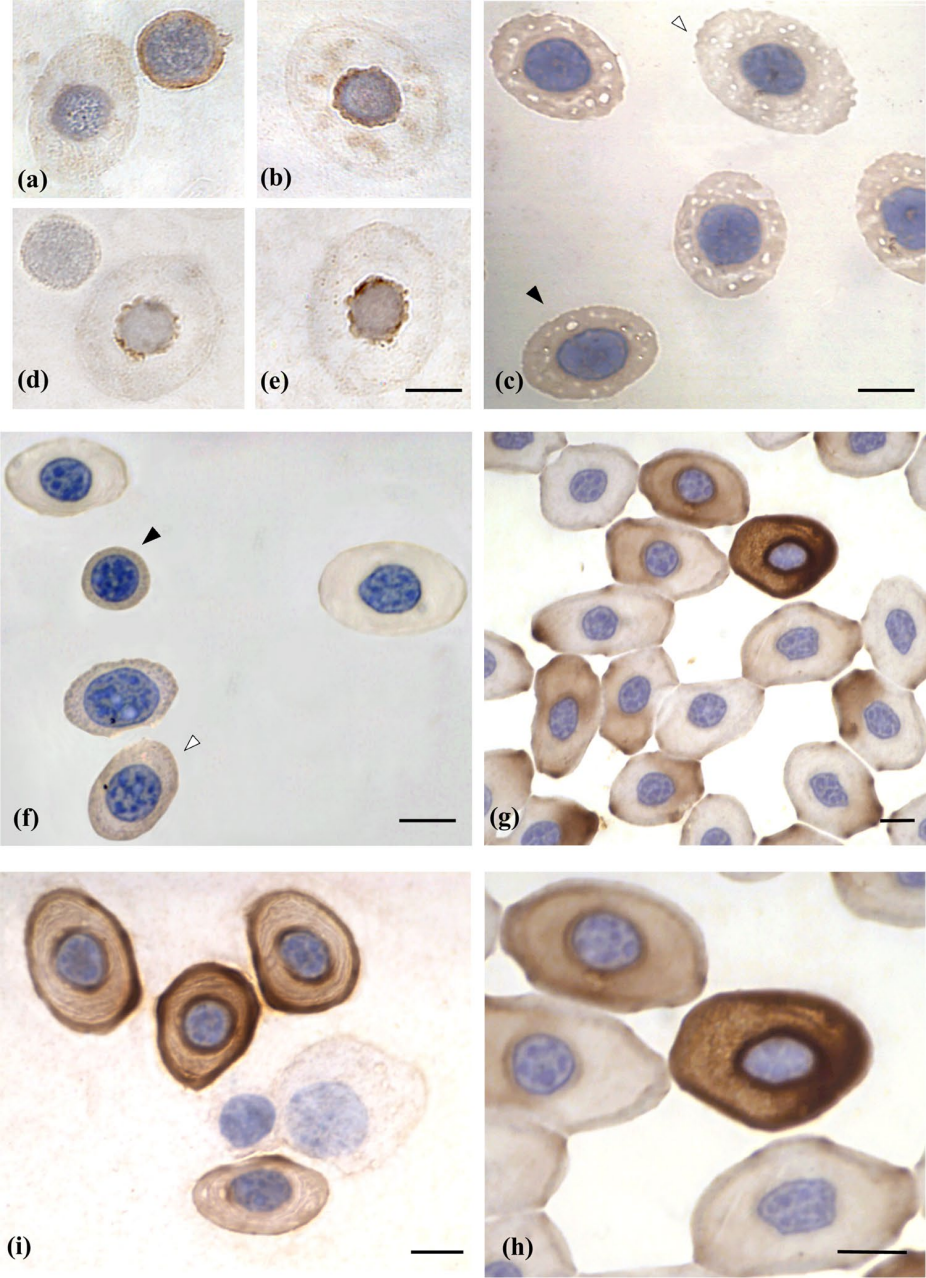
Light microscopy images of red blood cells from *Torpedo marmorata* Risso and *Caretta caretta*. (**a**) Bcl-2 protein is located in the cytoplasm of torpedo basophilic erythroblast. Scale bar as in **e**. (**b**) In the torpedo acidophilic erythroblast and mature erythrocyte, Bcl-2 and mitochondria have perinuclear position. Scale bar as in **e**. (**c**) Bcl-X_L_ is slightly detected in the torpedo acidophilic erythroblast (black arrowhead), but not in the mature erythrocyte (white arrowhead). Scale bar: 15 µm. (**d**,**e**) Bax immunolabeling is shown in torpedo acidophilic erythroblast (**d**) and mature erythrocyte (**e**). No Bax immunolabeling was showing in basophilic erythroblasts. Scale bar: 15.4 µm. (**f**) Demonstration of Bcl-2 immunopositivity in the turtle basophilic erythroblast (black arrowhead) and in the reticulocyte (white arrowhead). The reactivity is seen to decrease in the acidophilic erythroblast and the mature erythrocyte. Scale bar: 12 µm. (**g**) Bcl-X_L_ is detectable in the acidophilic erythroblasts. Scale bar: 12 µm. (**h**) Detail of panel of G showing the intense Bcl-X_L_ positivity of an acidophilic erythroblast, near a negative mature erythrocyte. Scale bar: 17 µm. (**i**) Bax is absent in immature turtle stages, while in mature stages it is localized in the cytoplasm. Scale bar: 15 µm.

The immunocytochemical reactions in light microscopy reveal a diffuse immunopositivity to Bcl-2 protein in the cytoplasm of torpedo basophilic erythroblasts. In the subsequent maturational stages, i.e., the acidophilic erythroblast, the immunopositivity reacts lightly in the mitochondria. They display a perinuclear disposition following staining Janus green, as demonstrated by Pica et al*.*^6^ (Fig. 1a,b).

The immunopositivity to Bcl-X_L_ protein is revealed only slightly and solely in the cytoplasm of the acidophilic erythroblasts, and not in mature erythrocytes (Fig. 1c).

Conversely, Bax is expressed as an intense immunopositivity in the mitochondria of the mature acidophilic erythroblast and mature erythrocyte. No Bax reactivity is detected in basophilic erythroblasts (Fig. 1d,e). No differences have been observed between the two species of torpedoes.

Immunocytochemical reactions performed on turtle blood smears reveal a diffuse immunopositivity to Bcl-2, which decreases until it disappears in the mature erythrocyte (Fig. 1f). Bcl-X_L_ protein is detectable in the cytoplasm of acidophilic erythroblasts, displaying various intensity, while it is lacking in mature erythrocytes (Fig. 1g,h). On the other hand, Bax is absent in basophilic erythroblasts and progressively increases in acidophilic erythroblasts and mature erythrocytes (Fig. 1i).

### X-ray damage: effects on protein expression in Torpedo erythrocytes

**Figure 2:**
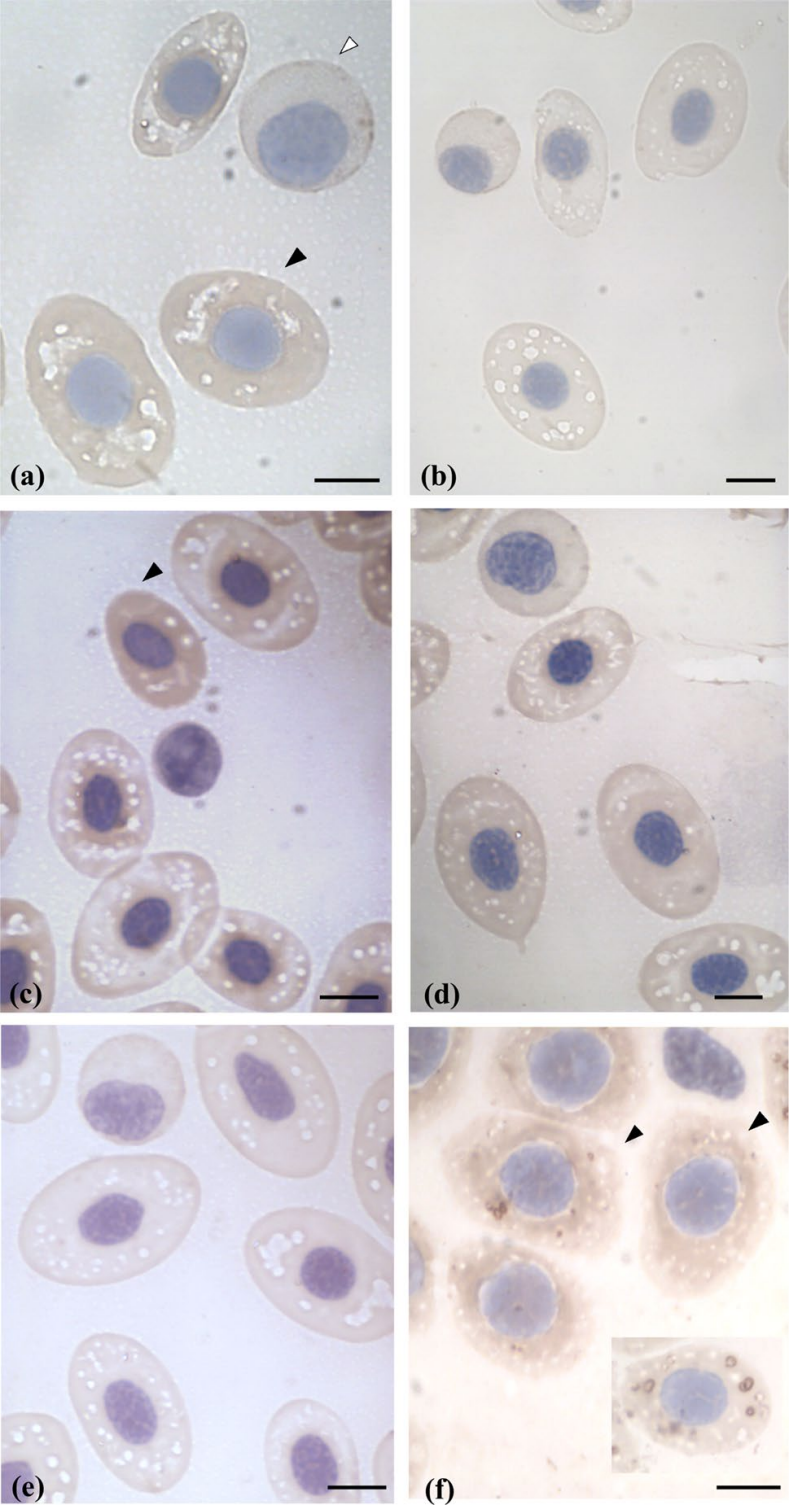
Light microscopy images of red blood cells from Torpedo after 40 Gy irradiation and after 90 Gy irradiation. (**a**) After 40 Gy irradiation we observed a weakly Bcl-2 immunopositivity in basophilic erythroblasts (white arrowhead) and in acidophilic erythroblast (black arrowhead). (**b**) After 90 Gy irradiation, no Bcl-2 immunopositivity was detected in all three stages of maturation. (**c**) After 40 Gy irradiation, Bcl-X_L_ immunopositivity was detected in the acidophilic erythroblast (black arrowhead). (**d**) After 90 Gy irradiation, a weakly Bcl-X_L_ immunopositivity was observed. (**e**) After 40 Gy irradiation, no Bax immunopositivity was observed. (**f**) After 90 Gy irradiation, Bax was identified in the acidophilic erythroblast (black arrowhead) and in the mature erythrocyte (white arrowhead). In the box, a young erythrocyte displaying Bax positivity in mitochondria. Scale bar: (**a**) 15.5 µm, (**b**,**f**) 9 µm, (**c**,**e**) 11.5 µm, (**d**) 15 µm.

In torpedoes irradiated at 40 Gy, a Bcl-2 weak positivity was detected in basophilic erythroblasts and in acidophilic erythroblasts (Fig. 2a). Bcl-X_L_ positivity was revealed only in acidophilic erythroblasts (Fig. 2c). No Bax positivity was found in erythroblasts or mature erythrocytes, after 40 Gy irradiation (Fig. 2e).

After higher radiation doses (90 Gy), no Bcl-2 immunopositivity was detected in all three stages of maturation (Fig. 2b). A very weak Bcl-X_L_ positivity was observed in developing or mature erythrocytes (Fig. 2d).

The Bax expression was only revealed in acidophilic erythroblasts and in mature erythrocytes of torpedoes irradiated at 90 Gy. In the box, a young erythrocyte displaying Bax positivity in mitochondria (Fig. 2f).

### Immunocytochemical findings in electron microscopy

**Figure 3:**
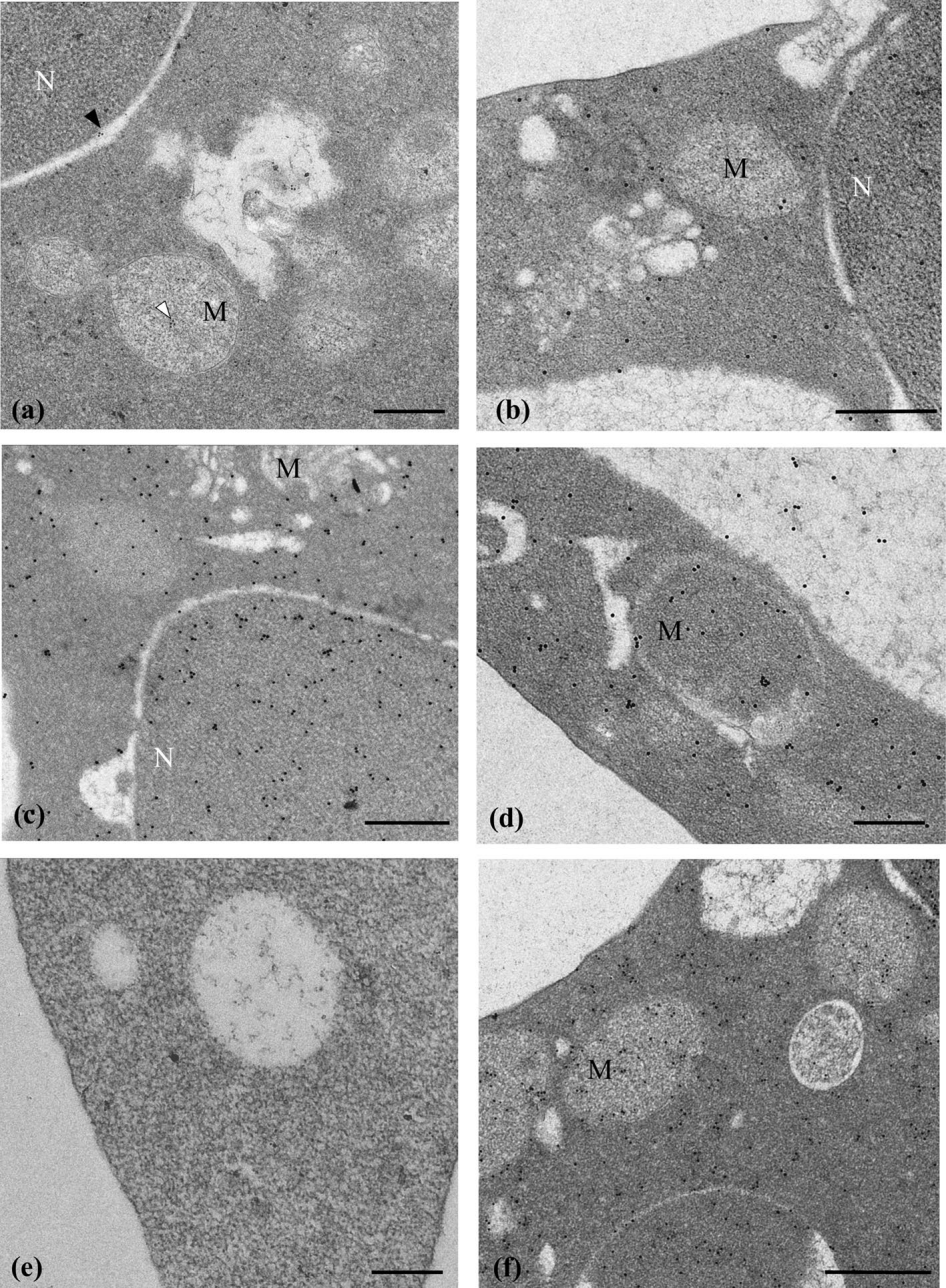
Electron microscopy images of red blood cells from Torpedo. (**a**) Immunoelectron microscopy reveals the Bcl-2 positivity, as indicated by colloidal gold particles (10 µm diameter), in the mitochondrial matrix (white arrowhead) and nuclear membrane (black arrowhead) of erythroblasts. (**b**) After 90 Gy irradiation, the localization of the Bcl-2 protein remains unchanged and its expression appears slightly reduced. (**c**) Bcl-X_L_ positivity was detectable by numerous and dispersed gold colloidal particles in cytosol of mature red blood cells. (**d**) Bcl-X_L_ positivity: some gold particle aggregates appears to be dispersed in cytosol after 90 Gy irradiation. (**e**) Weak Bax reactivity is identified in the cytosol of mature erythrocytes. (**f**) An increased expression of Bax was observed in mitochondria and in cytosol after 90 Gy X ray in erythrocytes. Scale bar: 0.5 µm. *M* mitochondria; *N* nucleus.

**Figure 4:**
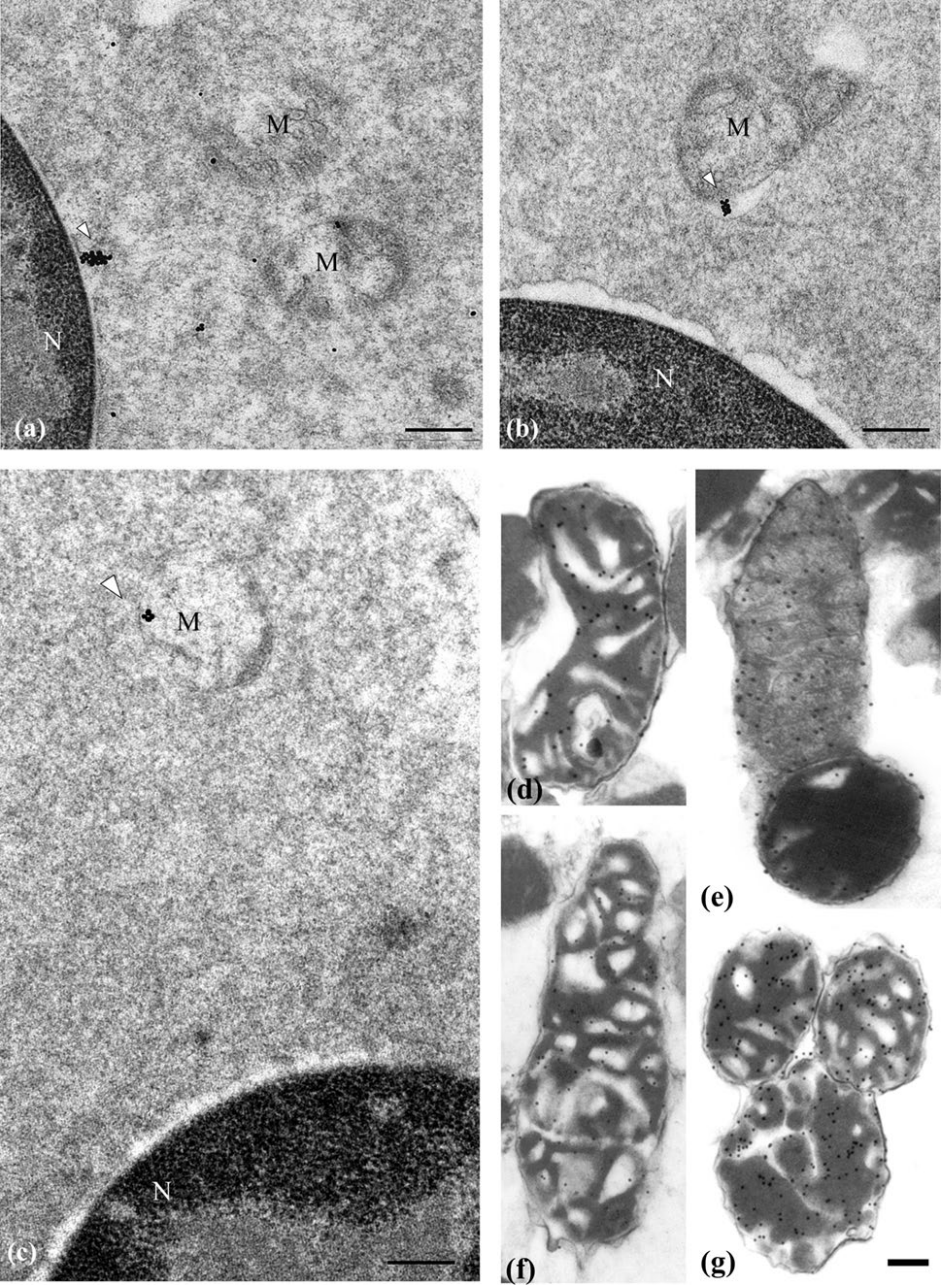
Electron microscopy images of mitochondria of *Caretta caretta* and of *Torpedo marmorata* Risso. (**a**) Loggerhead erythroblast immunolabeling with Bcl-2 antibody shows the location of Bcl-2 (25 nm colloidal gold) on the nuclear membrane (white arrowhead), cristae of the inner mitochondrial membrane and in the cytosol. (**b**) Bcl-X_L_ immunopositivity (25 nm colloidal gold) is observed on the internal mitochondrial membrane (white arrowhead) of young erythrocyte. (**c**) Bax is detected on the crest of a mitochondrion (white arrowhead) in mature erythrocyte. Scale bar: 0.5 µm. (**d**,**e**) Illustrations of Bcl-2 immunopositivity located on the internal mitochondrial membrane of torpedo young erythrocyte. (**f**,**g**) Bax positivity is observed to be mainly dispersed on the internal crests of the mitochondria of mature erythrocyte. Scale bar: 0.3 µm. *M* mitochondria, *N* nucleus.

**Figure 5:**
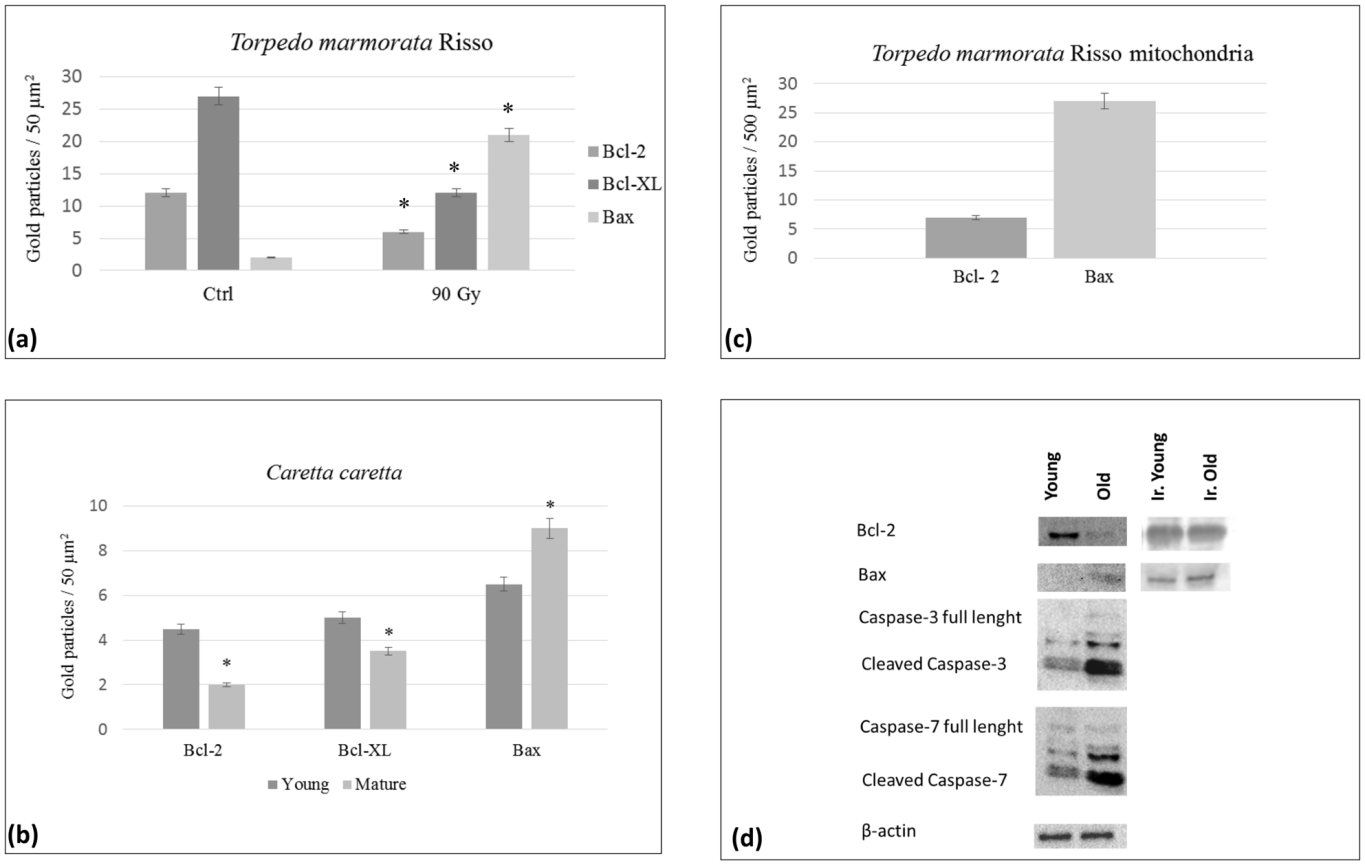
Quantifying immunogold labelling in transmission electron microscopy and western blotting. (**a**) In torpedo erythrocytes a significant decrease of Bcl-2 and Bcl-X_L_ and a significant increase of Bax were detected after 90 Gy irradiation (p < 0.5). (**b**,**c**) Quantifying immunogold labelling in young and in old loggerhead erythrocytes and in mitochondria of *Torpedo marmorata* Risso*.* A significant increase of Bax and a significant decrease of Bcl-2 and Bcl-X_L_ were detected in old loggerhead erythrocytes, as compared to young cells (p < 0.5), in agreement with light microscopy images. (**d**) Western blotting showing apoptotic proteins involved in erythropoiesis in young and old Torpedo erythrocytes. Bcl-2 was expressed mainly in young erythrocytes. On the contrary, Bax was detected in mature red cells. After irradiation, young erythrocytes (Ir. Young) and mature erythrocytes (Ir. Old) express both Bax and Bcl-2. Moreover, cleaved caspases-3/7 were expressed at higher levels in mature erythrocytes, compared to young ones. The β-actin was used as control.

The immunocytochemical analysis of ultrafine sections of unirradiated torpedo erythrocytes shows that the anti-apoptotic protein Bcl-2 is mainly located in the cytosol but it is also present on the nuclear membrane as well as in the mitochondrial matrix (Figs. 3a, 4, 5a) of erythroblast. In particular, this protein, as indicated by colloidal gold particles, is distributed on the crests of torpedo’s mitochondria and not on the inner mitochondrial membrane (Figs. 4d,e, 5c).

In the erythrocytes of torpedo that have been irradiated at 90 Gy, Bcl-2 displays a predominantly cytosolic and mitochondrial localization (Figs. 3b, 4, 5a).

The anti-apoptotic protein Bcl-X_L_ is localized in cytoplasm of unirradiated torpedo mature red blood cells while post-irradiation there is a decrease in cytoplasmic expression of this protein (Figs. 3c,d, 4, 5a).

The expression of the pro-apoptotic protein Bax is weakly detectable in the cytosol of mature erythrocytes of unirradiated torpedoes and appears mostly on the crests of the mitochondria of the acidophilic erythroblasts and mature erythrocytes (Figs. 3e, 4f,g, 5c). In regard to the 90 Gy-irradiated torpedoes, their erythrocytes offer an intense positivity of the Bax reaction in the cytoplasm and mitochondria (Figs. 3f, 4, 5a).

Immunocytochemical reactions performed on loggerhead erythroblast revealed Bcl-2 and Bcl-X_L_ positivity on the nuclear membrane, inner-mitochondrial cristae intercalated by flat inner-mitochondrial membrane and in the cytosol of young erythrocyte (Figs. 4a,b, 5b). Bax was detected on the crest of the mitochondria of acidophilic erythroblast and mature erythrocyte (Figs. 4c, 5b).

### Apoptotic protein expression in young and old Torpedo erythrocytes

The western blotting shows the protein bands detected with anti-Bax and anti-Bcl-2 antibodies after electrophoretic separation of proteins of torpedo immature and mature erythroid cells. Bcl-2 was expressed mainly in young erythrocytes. Contrarily, Bax was detected in mature red cells. Moreover, cleaved caspases-3/7 were expressed at higher levels in mature erythrocytes, compared to young ones. After irradiation, young and mature erythrocytes express both Bax and Bcl-2 (Fig. 5d).

## Discussion

In the unusual dichotomy, i.e., mammalian versus poikilothermic species’ red cells, the fully developed mature mammalian red blood cell has extruded its nucleus and has degraded and released most of its metabolic machinery so that the cell is devoid of the metabolic capability to enter apoptotic activity. Such cells have often characterized as plastids.

This exceptional well recognized status generates multiple questions. Do the erythrocytes that permanently retain their nuclei and maintain at least most of their metabolic machinery, i.e., the red blood cells of poikilotherms, enlist the same metabolic pathways observed in apoptotic somatic cells observed in all species? Apoptotic activators and apoptotic inhibitors have been identified in mammalian non-erythroid somatic cells. Is this molecular interaction identifiable in the permanently nucleated red cells of the poikilotherm? Is it identifiable in the erythrocytes of the earliest evolutionary vertebrates (the elasmobranchs)? If this is indeed the case, is this capability conserved in further developed poikilothermic species? This present investigation is designed to address some of these questions.

In fishes, erythropoiesis is significantly dependent on environmental conditions (principally temperature and level of oxygen). Therefore, seasonal changes in the level of erythrocytes in the circulating blood occur. The availability of oxygen can vary considerably in an aquatic environment and fish react to hypoxic conditions with an enhanced rate of erythropoiesis^12^.

As previously demonstrated, sublethal x-irradiation of torpedoes followed by autohemotransplant fully restored erythropoiesis only on red pulp of the spleen^11^. Moreover splenectomy of torpedoes induced circulating erythropoiesis to restore the physiological number of circulating red blood cells^13^. In another species, the dogfish *Scyliorhinus canicula*, Fänge and Johansson-Sjöbeck (1975) did not identify erythropoiesis in lymphomyeloid tissues after splenectomy; nevertheless they hypothesized that early erythropoiesis could take place in lymphomyeloid tissues (although they could not detect this activity) and complete the erythrocytic maturation in the circulating blood^14^.

The expression of some apoptotic proteins was investigated in this study to assess their involvement in the regulation of poikilothermic vertebrate erythropoiesis. The results indicate that these proteins, both anti-apoptotic and pro-apoptotic, regulate erythropoietic equilibrium in *Torpedo marmorata* Risso and in *Caretta caretta* (loggerhead) turtles.

The immunocytochemical analysis of pro- and anti-apoptotic proteins of torpedo erythrocytes reveals that both appear on the internal membrane of mitochondria.

Anti-apoptotic and pro-apoptotic proteins seem to have different expression over time. Bcl-2 and Bcl-X_L_ are identifiable in the juvenile stage of erythrocytes and its presence decreases in the subsequent maturational stage which is characterized by a decrease of RNA transcription, responsible for the diminished cytoplasmic basophilia of the further developed antecedent of the red cell.

Concomitantly, Bax expression increases. The Bax protein of the torpedo erythrocytes is expressed in the cytosol of the most mature erythroblast but in the further developed stages it is associated with the mitochondrial crest membrane.

These immunochemical data clearly demonstrate an increase in the expression of Bax and a corresponding decrease of Bcl-2 during erythropoiesis. In immature stages of erythroid cells, the higher Bcl-2 and Bcl-X_L_ expressions prevent apoptosis, meanwhile the increased Bax expression in maturative stages trigger the apoptotic process in the senescent cells. In fact, some apoptotic mechanisms are spontaneously activated for erythroid maturation e.g., the caspase activation during late stages of erythroid maturation^15^. Caspase-3/7 are the main apoptosis executioner and regulate mitochondrial events in the apoptotic pathway. Apoptosis can be also induced by the activation of caspase-7 that may partially substitute caspase-3 in caspase-3–deficient models^16^.

Caspase-3 is considered a reliable marker for cells that are dying, or have died by apoptosis^17^. Caspase-3 is a well executioner of eryptosis. Yange et al*.* demonstrated the role of caspase-3, as executioner of erythropoietin regulator apoptosis^18^.

On the other hand, during mammalian erythropoiesis, the origin of reticulocyte is due to trigger of apoptosis through caspases-3/7, which lead to the nucleus expulsion. This apoptotic process is not fully executed because of the exhaustion in caspase-3/7^19^.

The caspase-3 sequence appears to be evolutionary conserved in *Pacific cod* by showing a prominent role as mediator of apoptosis^20^. The complete sequence of caspase-3 of sea bass cells (*Dicentrarchus labrax* L) shows a very close homology to the correspondent sequence from other vertebrates^21^.

Caspase-3 has been demonstrated to be involved even in invertebrate cell apoptosis, as regulator of aging in *Daphnia pulex*^22^, or associated with degenerative processes in various tissues in four species of Molluscs^23^.

Otherwise, in the present study, caspase-3/7 activation was demonstrated in mature torpedo erythrocytes compared to young ones; activation of caspase-3/7 that act further downstream and direct cellular breakdown through cleavage of structural proteins is a hallmark of apoptosis. In our case, the activation of caspases-3/7 occurred in parallel with the increase of proapoptotic protein Bax and the decrease of antiapoptotic protein Bcl-2 by suggesting the activation of apoptotic pathway.

The study of irradiated torpedoes was conducted to assess the activation of pro- and anti-apoptotic proteins following X-ray exposure. A comparable investigation of *Caretta caretta,* however, was not carried out because it is a protected species.

As for the erythroid cells of irradiated torpedoes, the expression of anti-apoptotic proteins (Bcl-2 and Bcl-X_L_) is inhibited and the expression of the pro-apoptotic proteins (Bax) is increased, after 90 Gy irradiation. The observations derived from electron microscopy revealed a weak Bcl-2-positivity after X-ray exposure, that was not detectable under light microscopy. This status confirmed the information derived from immunoblotting. X-ray damage may alter physiological intracellular balance between pro-apoptotic and anti-apoptotic proteins. It is further of interest that the torpedo is particularly resistant to x-irradiation, very likely due to the known high amount of antioxidant molecules in their tissues^24^. As demonstrated in prior studies, the sublethal dose for the torpedo is 100 Gy while the lethal dose for humans is 7 Gy^10,11^. Peslak et al*.* demonstrated that the pro-erythroblast population of mice underwent radiation-induced apoptosis following 1 Gy total body irradiation^25^. This dose is almost the threshold of sub-lethal dose for mammals. Moreover many authors demonstrated that elasmobranchs have a lower incidence of neoplasia than that of any other vertebrate group^26^. Interesting applications of these features are the study of compounds produced by immune tissues of elasmobranch fishes, which can be used for development of new antibiotics and novel treatments for cancer, macular degeneration, viral pathogens, and Parkinson’s disease^27,28^. These observations suggest that further studies in this area should generate substantive results.

The present investigation of the sea turtle *Caretta caretta* reveals that anti-apoptotic and pro-apoptotic proteins appear to be involved in the maturation of erythroid cells. Diffuse immunopositivity of both Bcl-2 and Bcl-X_L_ is obtained in the cytoplasm of the earliest precursor of the erythrocyte and this reactivity weakens thereafter. Conversely, Bax, not identifiable in the basophilic erythroblasts, is evident in the subsequent stages.

In conclusion, Bcl-2 sub-family is involved in regulation of erythropoiesis in non-mammalian vertebrates. Hence, apoptotic mechanism appears to be conserved among the species that maintain permanently nucleated erythrocytes regardless of the evolutionary interval among them (see also^29^).

## Methods

### Animals and blood samples

Juvenile and adult torpedoes (two specimens of *Torpedo marmorata* Risso, one male and one female, seven specimens of *Torpedo ocellata* Rafinesque, two male and two female, weighing 500–600 g, and three samples, two female and one male, weighing 300 g), six specimens (2 T*. marmorata* and 4 T*. ocellata*) used as controls, and 20 specimens of loggerhead turtles *Caretta caretta* (weighing between 1.33–53.78 kg, 8 male and 12 female) were caught in the bay of Naples and in the Adriatic and Tyrrhenian Seas, were kept in suitable-sized tanks filled with flowing sea water at 18–20 °C and regularly fed fresh fish and mollusks at the zoological station of Naples. All animals used in the present study were healthy, as confirmed by hemogram performed.

All torpedoes were similar in weight; they were divided in two groups (treated and controls) by species and sex.

Torpedo blood samples were quickly taken from the caudal vessel by using a 2.5 mL syringe and collected into tubes containing the anticoagulant EDTA and thin blood smears for light microscopy were made immediately with non-anticoagulated blood. The blood was centrifuged at 1000 rpm for 15 min and two erythrocyte fractions were separated: the immature erythrocyte-rich level (under the discarded buffy coat layer of the red cell column) and the mature red cells (the deep region of the red cell column). The two samples were then subjected to lyophilization and used for subsequent assays.

Turtle blood samples were quickly drawn from the dorsal occipital venous sinus into evacuated tubes containing 0.1% lithium heparin (BD, Buccinasco, Milan, Italy). Stabulated turtles actively collaborated to blood withdrawal, by lowering their heads, waiting for the prize fish following blood collection. Thin smears were made immediately from non‐anticoagulated blood and cytochemical reactions were performed.

Fish care, experimental protocols and all methods were performed in accordance with relevant guidelines and regulations. The study was carried out in compliance with the ARRIVE guidelines and was approved by the “Committee for the Protection of Animals used for Experimental and other Scientific Purposes of the University of Naples Federico II”.

### Torpedo irradiation

Torpedoes were irradiated at 40 and 90 Gray (Gy) using the linear accelerator (Siemens) to study the changes induced by radiation on the apoptotic mechanisms that regulate erythropoiesis. The animals were kept in the tank with circulating sea water and after seven days post-irradiation and subsequently once a week blood samples were taken from the caudal vein, as described, to perform hemogram, blood smears and the described methods.

### Immunocytochemistry at light microscopy

The blood smears were fixed in 4% paraformaldehyde for 2 min and then washed in distilled water. The high temperature antigen unmasking technique using citrate buffer was performed. A microwave oven (MW 310 DeLonghi,Treviso, Italy) was used. The blood smears were submerged in 10 mM citrate buffer (pH 6.0) and microwaved at 400 W 2 treatments 2 times for 5 min. Subsequently, the smears were left in the citrate buffer at room temperature for 20 min.

The blood smears were washed and reacted for 15 min in 3% H_2_O_2_ to inactivate endogenous peroxidase activity and incubated for 60 min at room temperature in 5% normal goat serum (NGS; Dako, Glostrup, Denmark) in 0.1 M phosphate-buffered saline pH 7.4 (PBS), containing 0.1% Triton X-100 (Sigma, St. Louis, MO). The smears were then incubated overnight, in a humid chamber, in rabbit polyclonal antibodies that recognize Bcl-2 (Santa Cruz Biotechnology, Santa Cruz, CA) diluted 1:100 in NGS. After several rinses, the smears were incubated for 2 h in biotinylated goat anti-rabbit IgGs (Vector Laboratories, Burlingame, CA) diluted 1:50 in NGS, followed by incubation for 1 h at room temperature in the avidin–biotin-peroxidase solution (ABC Kit; Vectastain, Vector) in PBS, and then for 10 min in 0.05% 3–3’diaminobenzidine (DAB) and 0.01% H_2_O_2_ in 0.01 M Tris–HCl-buffered saline, pH 7.6 (TBS). Negative control smears were processed with the same protocol omitting the primary antibody. No immunostaining was detected in these sections.

The same procedure was followed for Bcl-X_L_ and Bax immunostaining using rabbit polyclonal anti-Bcl-X_L_ and anti-Bax antibodies (Santa Cruz Biotechnology, Santa Cruz, CA; diluted 1:100 in NGS).

### Isolation of red blood cell mitochondria

Isolation of red blood cell mitochondria was described by Pica et al*.*^6^. Briefly, the red blood cells were centrifuged at 800*g* for 15 min to obtain a mitochondrial pellet which was then fixed for electron microscopy. The cell pellet was diluted three-fold with buffer solution (800 mM sucrose, 2 mM K-EDTA, 10 mM Tris, pH 7.4) and incubated with 200 µg/mL digitonin for 15 min at 4 °C. After a centrifugation at 8000*g*, 4 °C for 10 min, the pellet of Hb-free permeabilized cells was resuspended in the same initial volume of the previous buffer solution and incubated with 400 µg/mL lysozyme and 200 µg/mL nagarse for 20 min at 4 °C. This cell suspension was then disrupted at 0 °C with a Potter–Elvehjem homogenizer. The homogenate was centrifuged at 1500*g* and the supernatant collected by glass fiber filtration and centrifuged twice at 7000*g*. The mitochondrial pellet was resuspended, centrifuged at 22,000*g* for 10 min. Mitochondrial pellet was collected in the buffer solution at a concentration of 5–6 mg prot/mL. The protein concentration was determined by using the Bradford method.

### Isolation of mitoplasts

The red blood cells were centrifuged at 14000*g* for 10 min and resuspended in phosphate buffered saline (PBS) containing 5 mM NaF, 500 nM okadaic acid and 1 mM sodium orthovanadate as phosphatase inhibitors. The protein concentration was determined by using the Bradford method and digitonin (0.2 mg/mg of protein) was added to cell suspension. The sample was incubated in ice for 10 min and subsequently diluted five-fold in PBS and centrifuged at 10,000*g* for 10 min. The supernatant containing the cytosolic fraction was removed and the pellet of mitoplasts was re-suspended in PBS and re-centrifuged to remove digitonin residues, as described by Technikova-Dobrova et al*.*^30^.

### Immunoelectron microscopy

The red blood cell pellet and isolated mitochondria were fixed in 0.1% glutaraldehyde and 2% paraformaldehyde in 0.1 M sodium cacodylate buffer, for 30 min and washed in 0.1 M sodium cacodylate buffer for 1 h at 37 °C, postfixed with 1% OsO4 for 1 h, dehydrated in an ethanol-gradient, embedded in resin Epon 812 and polymerised for 24 h at room temperature and 24 h at 60 °C. For immunoelectron studies, ultrathin sections were processed according to a postembedding immunogold procedure. Ultrathin sections were achieved using a Reichert ultramicrotome and the sections were mounted on nickel grids.

The high temperature antigen unmasking technique was performed using citrate buffer and a microwave oven. The sections, having been placed on grids, were soaked in 10 mM citrate buffer, pH 6.0, in capped plastic jars and microwaved at 400 W (2 treatments for 5 min) and then the grids were left in the citrate buffer at room temperature for 20 min. The sections were incubated in 10% hydrogen peroxide for 10 min, rinsed in PBS for 15 min and blocked for 15 min in 1% bovine serum albumin (BSA) in PBS. The sections were then incubated with rabbit anti-Bcl-2, anti-Bcl-X_L_ and anti-Bax (Santa Cruz Biotechnology, Santa Cruz, CA) (dilution 1:50) overnight at 4 °C, followed by 25 nm colloidal gold labelled anti-rabbit secondary antibodies (Aurion, Netherlands, 1:20) for 2 h at room temperature. Sections were rinsed in PBS (pH 7.4) and distilled water prior to counterstaining with uranyl acetate and lead citrate. Ultrathin sections were examined in a Philips 400-TEM.

### Quantifying immunogold labelling in transmission electron microscopy

The quantification of immunogold labelling in transmission electron microscopy was detected by counting all gold particles per area unit (µm^2^), according to D’Amico et al*.*^31^. All values were expressed as means ± standard deviation of three different experiments (p < 0.5).

### Electrophoresis, immuno- and western- blotting

The pellets of mitoplasts and untreated mitochondria were re-suspended in lysis buffer (50 mM Tris-Cl, 15% glycerol, 2% β-mercaptoethanol, 5% sodium dodecyl sulphate, pH 6.8) and subjected to sodium dodecyl sulphate–polyacrylamide gel electrophoresis (SDS-PAGE) (12% polyacrylamide, 1% SDS). The separated proteins were subsequently electro-transferred into a nitrocellulose filter membrane, previously activated by immersion in a blotting buffer (200 mM glycine, 25 mM Tris, 20% methanol, 0.1% SDS) for 30 min.

After 1 h, the nitrocellulose filter membrane was washed in TTBS buffer (20 mM Tris/HCl, 0.5 M NaCl, 0.005% PBS-Tween, pH 7.5) and incubated overnight with primary polyclonal anti-rabbit antibody anti-Bax (Santa Cruz Biotechnology, Santa Cruz, CA; 1:100 in TTBS). After washing, the membrane was incubated with a conjugated goat anti-rabbit secondary antibody labeled with peroxidase (Vector, CA; 1:3000) for 1 h and washed in TTBS. The chemiluminescence was detected by ECL kit (Dupont/NEN, USA). The same protocol was used for the detection of Bcl-2 (polyclonal antibody anti-rabbit anti-Bcl-2—Santa Cruz Biotechnology, Santa Cruz, CA).

For the detection of caspases, proteins were extracted from cells by using RIPA Lysis Buffer (Millipore) and following the manufacturer’s instructions. Western blot was performed by 20 µg total protein (quantified using Bradford assay, Bio-Rad) on a 10% polyacrylamide gel and blotted onto PVDF membranes (Millipore, Marlborough, MA). Primary antibodies against Bax (#2772), Bcl-2 (#3498), caspase-3 (#9662) and caspase-7 (#9492) were purchased from Cell Signaling Technology Inc. (Beverly, MA, USA). The chemiluminescent system (ECL; Amersham Life Science, Buckinghamshire, UK) visualized protein bands. The membrane was captured using LI-COR System and quantified with Quantity One 1-D analysis software (Biorad Laboratories). Protein bands were normalized against β-actin level (# 4970, Cell signaling Technology).

### Ethics declarations

The experiments were performed under institutional approval and all efforts were made to avoid animal suffering.

## Supplementary Information

Supplementary Information 1.

Supplementary Information 2.

Supplementary Information 3.

Supplementary Information 4.
