# Supplementary figures and images for: Evolutionary conservation of a regulative pathway of erythropoiesis in Poikilothermic vertebrates
Source: Sci Rep. 2022 Feb 28;12:3307. doi: 10.1038/s41598-022-06617-6 (PMC8885823; doi:10.1038/s41598-022-06617-6)

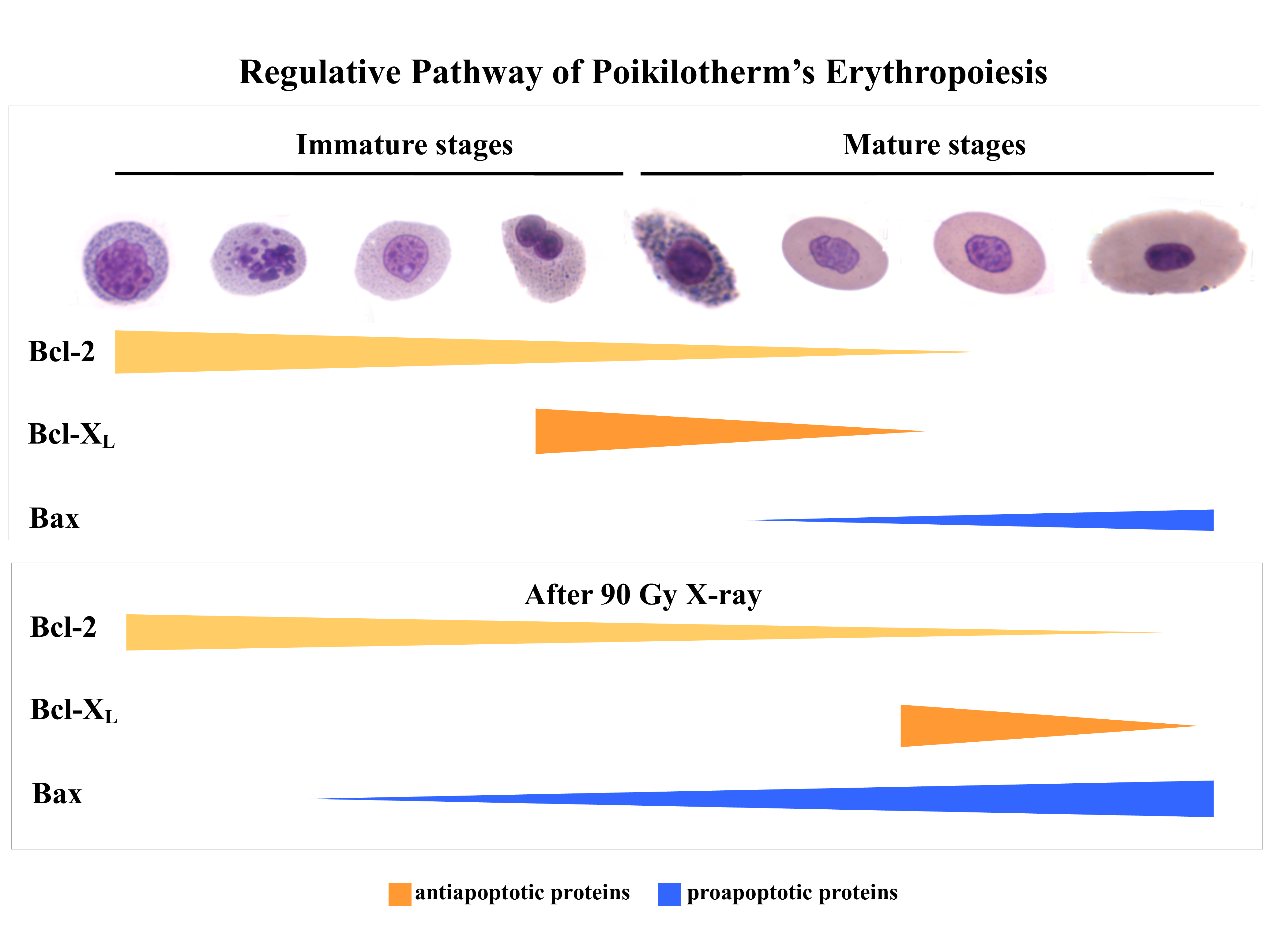

Supplement: Supplementary file 1 — Supplementary Information 1. [file 41598_2022_6617_MOESM1_ESM.tif]

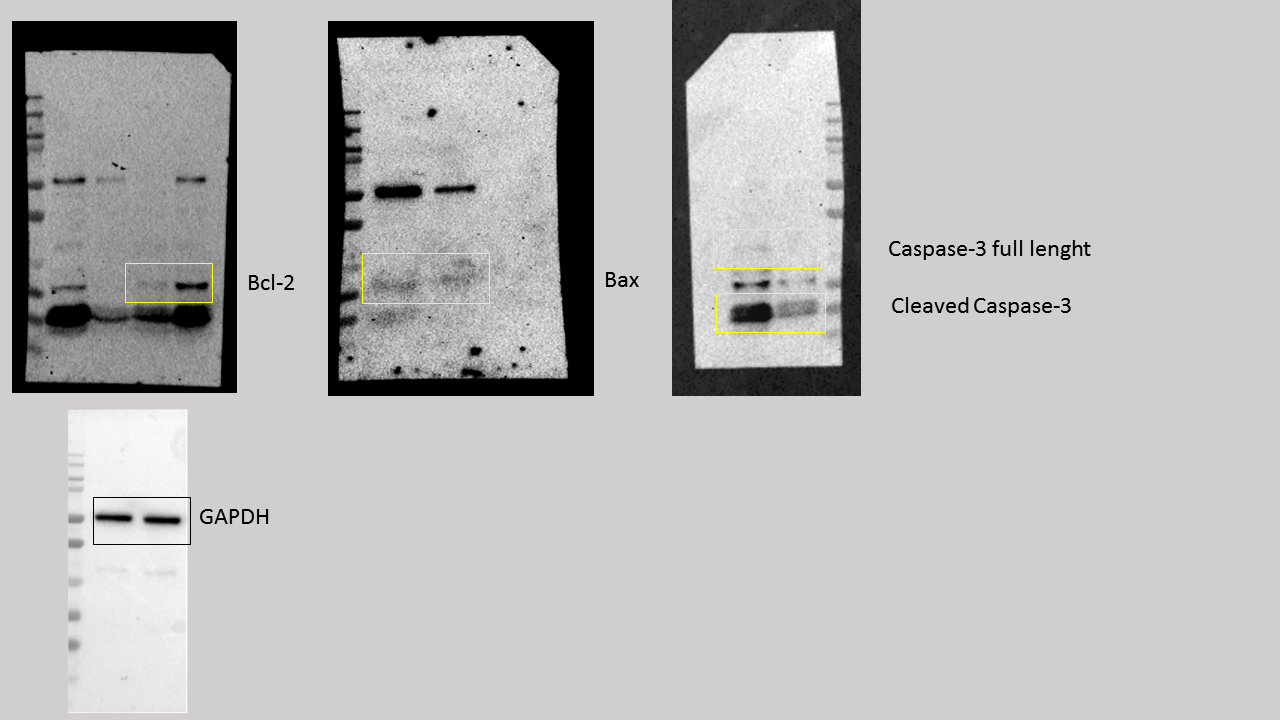

Supplement: Supplementary file 2 — Supplementary Information 2. [file 41598_2022_6617_MOESM2_ESM.tif]

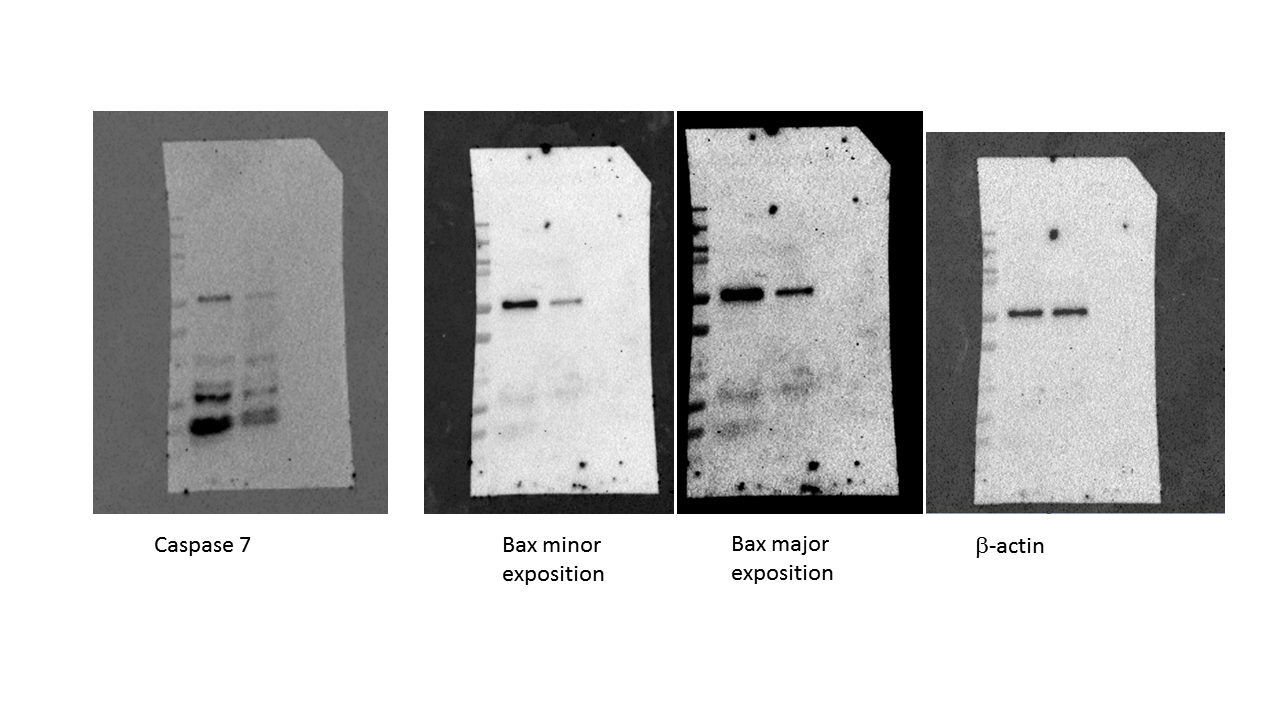

Supplement: Supplementary file 3 — Supplementary Information 3. [file 41598_2022_6617_MOESM3_ESM.tif]

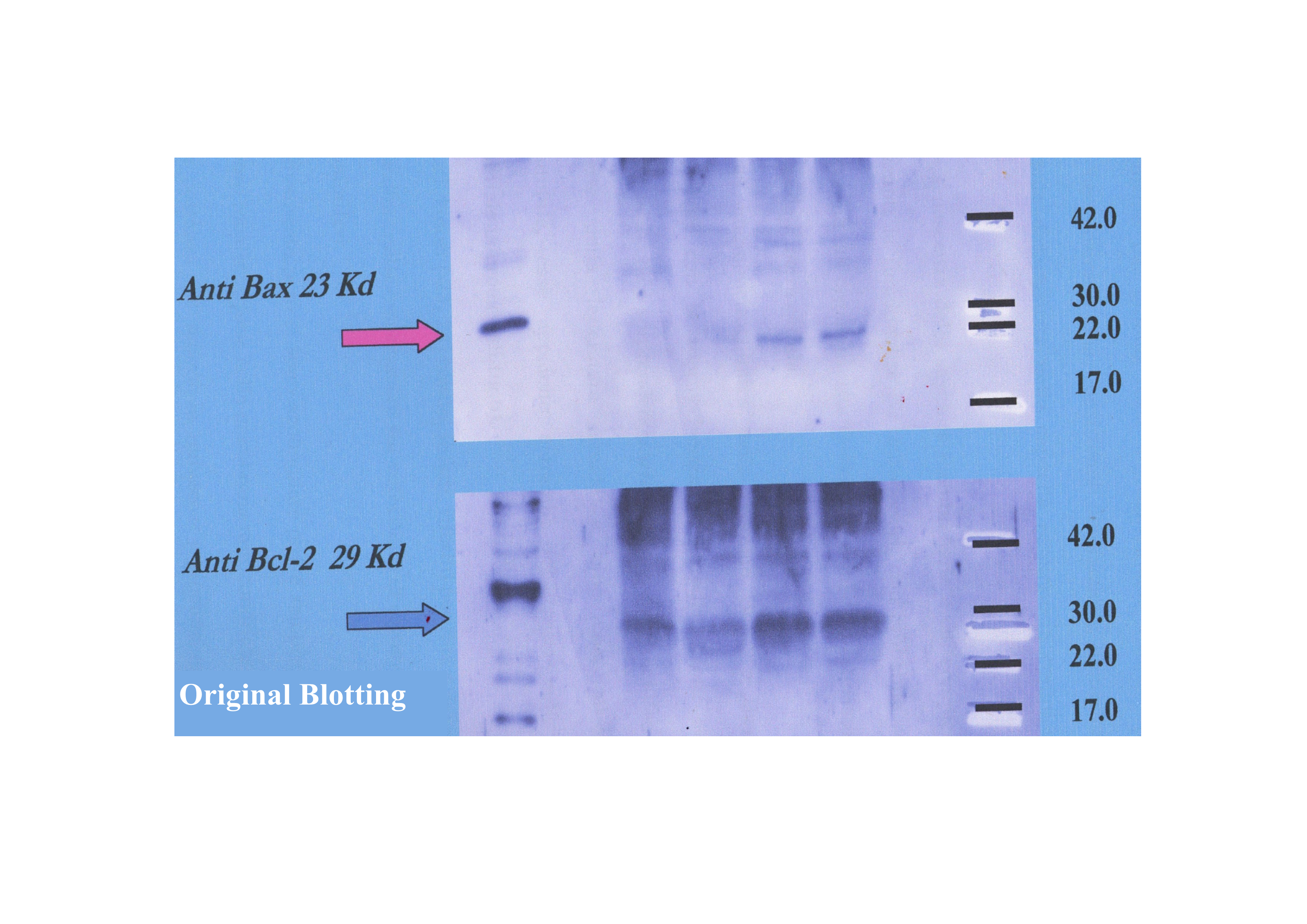

Supplement: Supplementary file 4 — Supplementary Information 4. [file 41598_2022_6617_MOESM4_ESM.tif]
